# Longitudinal Serum Neurofilament Levels of Multiple Sclerosis Patients Before and After Treatment with First-Line Immunomodulatory Therapies

**DOI:** 10.3390/biomedicines8090312

**Published:** 2020-08-28

**Authors:** André Huss, Makbule Senel, Ahmed Abdelhak, Benjamin Mayer, Jan Kassubek, Albert C. Ludolph, Markus Otto, Hayrettin Tumani

**Affiliations:** 1 Department of Neurology, University Hospital of Ulm, Oberer Eselsberg 45, 89081 Ulm, Germany; andre.huss@uni-ulm.de (A.H.); makbule.senel@uni-ulm.de (M.S.); ahmed.abdelhak@ucsf.edu (A.A.); jan.kassubek@uni-ulm.de (J.K.); Albert.Ludolph@rku.de (A.C.L.); markus.otto@uni-ulm.de (M.O.); 2 Department of Neurology and Stroke, University Hospital of Tübingen, Hoppe-Seyler-Alle 3, 72076 Tübingen, Germany; 3 Hertie institute of clinical of clinical brain research, University of Tübingen, Hoppe-Seyler-Alle 3, 72076 Tübingen, Germany; 4 Institute of Epidemiology and Medical Biometry, Ulm University, Schwabstraße 13, 89075 Ulm, Germany; benjamin.mayer@uni-ulm.de; 5 Speciality Clinic of Neurology Dietenbronn, Dietenbronn 7, 88477 Schwendi, Germany

**Keywords:** multiple sclerosis, serum neurofilament, immunomodulatory therapies, therapy-response marker

## Abstract

Serum neurofilament light chain (NfL) has been shown to correlate with neuroaxonal damage in multiple sclerosis (MS) and various other neurological diseases. While serum NfL is now regularly reported in clinical approval studies, there is a lack of longitudinal data from patients treated with established basic immunotherapies outside of study conditions. In total, 34 patients with early relapsing-remitting MS (RRMS) were included. The follow-up period was 24 months with regular follow-up visits after 3, 6, 9, 12 and 18 months. Therapy with glatiramer acetate was initiated in 20 patients and with interferon-beta in 12 patients. The disease course was monitored by the events of relapses, Expanded Disability Status Scale (EDSS) score and MRI parameters. Overall, serum NfL levels were higher at time points with a current relapse event than at time points without relapse (12.8 pg/mL vs. 9.7 pg/mL, *p* = 0.011). At follow-up, relapse-free patients showed significantly reduced serum NfL levels starting from 9 months compared to baseline (*p* < 0.05) and reduced levels after 12 months compared to baseline (*p* = 0.013) in patients without EDSS progression for 12 months. In this explorative observational study, our data suggest that the longitudinal measurement of serum NfL may be useful in addition to MRI to monitor disease activity and therapy response.

## 1. Introduction

Multiple sclerosis (MS) is a chronic inflammatory disease of the central nervous system (CNS) characterized by demyelination and axonal loss [1]. The current concept of MS pathology is based on infiltrating immune B- and T-cells via the blood–brain barrier, local antibody production and activation of glial cells [2,3]. These processes are thought to lead to primary demyelination followed by neurodegeneration [2]. In recent years, several neurochemical markers have been established for the characterization of pathological molecular processes. One of the most extensively investigated markers for neuroaxonal loss is neurofilament light chain (NfL) [4,5,6]. NfL is one of four neurofilament subunits and the most abundant one, making it a popular target for neurological diseases [7]. Here, NfL showed superior sensitivity for MS than the phosphorylated subunit of neurofilament [8].

Initially investigated using standard immunoassays, NfL in the cerebrospinal fluid (CSF) from MS patients was found to correlate with disease course and activity [9,10]. In the early phase of the disease, it has a prognostic value [5,11,12] and can be used as a treatment response marker [13]. However, NfL is not specific for MS, is rather a general marker for neurodegenerative processes [14,15] and changes with the normal aging brain [16], which needs to be considered when looking at NfL changes over time.

As detection methods were developed over the years, highly sensitive immunoassays became available and allowed the analysis of brain-derived proteins, not only in the CSF, but in serum as well [17]. Beyond showing a good correlation with CSF values, serum NfL has already thoroughly been investigated in MS [18], i.e., it has been shown to correlate with clinical and radiological disease activity (relapses, new/enlarged T2 lesions and gadolinium-enhancing lesions in magnetic resonance imaging (MRI)) [19,20,21,22]. The most important advantage of serum analyses is the possibility of serial sampling and consecutive analysis of biomarkers. Thus, NfL is regularly used in clinical trials to monitor therapy efficacy, and it is on the footsteps of being used as a secondary outcome parameter in clinical trials [23].

In most studies, group effects of treatments on neurofilaments are investigated, which already indicate the applicability of serum NfL as a therapy response marker [22,24] and as a prognostic marker for long-term clinical outcomes in MS [25]. However, longitudinal data of intraindividual NfL levels over disease course under immunomodulatory therapies in well-characterized MS patients are widely missing and only described rarely [26,27].

In this study, we analyzed consecutive samples of MS patients in the early phase of the disease, before and after the initiation of disease-modifying treatment with either glatiramer acetate or interferon-beta over a follow-up period of 24 months. Serum NfL levels at each visit were correlated to clinical outcome parameters (relapse and Expanded Disability Status Scale (EDSS)), serum cytokine profile, cognitive functions and MRI parameters of disease activity and progression.

The aim of this study was (a) to show the effect of immunomodulatory therapies on serum NfL levels in MS patients over disease course, (b) to evaluate the relationship between NfL and MRI parameters reflecting disease progression, such as T2 lesion load, (c) to evaluate possible correlation with cognitive functions and (d) to compare serum NfL levels with the serum cytokine profile.

## 2. Experimental Section

### 2.1. Patients

In total, 34 patients who attended the Department of Neurology at the University Hospital Ulm between 2002 and 2004 before initiation of disease-modifying treatment (DMT) were included in the study. Initially, the MS diagnosis was made on the diagnostic criteria valid at time of study inclusion (McDonald 2001), but were adjusted for the most recent updates of the McDonald criteria (McDonald 2017). After study inclusion, 20 patients started treatment with glatiramer acetate, 12 patients were treated with interferon-beta (Avonex, Betaferon and Rebif) and 2 patients rejected DMT. All patients were then followed-up for 24 months with visits every 3 months in the first year and every 6 months in the second year. At all visits, clinical assessments including relapse evaluation, EDSS, Paced Auditory Serial Addition Test (PASAT), and serum sampling were performed. Relapses were defined as focal neurological disturbance lasting more than 24 h, without an alternate explanation. Furthermore, 17 patients received magnetic resonance imaging (MRI) scans at baseline, 12 months and 24 months. Detailed patients’ characteristics are shown in Table 1 and the study schedule is shown in Table 2. Relapses were treated with high-dose corticosteroids (50–1000 mg) over 3–5 days after exclusion of contraindications. Age did not differ between patients with and without at least one relapse during follow-up and did not correlate with serum NfL levels at baseline.

### 2.2. NfL Measurements

Serum samples were stored in the local biobank according to recommended biobanking protocols at −80 °C [28]. Serum NfL was measured using the Simoa technology (Quanterix Corporation, Lexington, MA, USA). Samples were diluted, as recommended by the manufacturer, and concentrations were calculated using the corresponding standard curve.

### 2.3. Cytokines Measurements

Cytokine profiles including IFN-γ, osteopontin (OPN), IL-2, IL-4 and IL-10 were determined in serum at study onset and at every visit during follow-up using the electrochemiluminescence detection multiplex technology of Meso Scale Discovery (MSD, Gaithersburg, MD, USA) according to the manufacturer’s instructions as previously reported [29].

### 2.4. MRI Scans

MRI scans of the brain and spinal cord were performed on a 1.5 Tesla clinical MRI scanner (Symphony Siemens, Erlangen, Germany) and the total number of hyperintense lesions in T2-weighted scans at the different time points were visually quantified by an experienced rater.

### 2.5. Cognitive Functions

Cognitive functions were assessed at every time point by the Paced Auditory Serial Addition Test (PASAT). Here, information processing speed and flexibility, as well as calculation ability are tested, which also means that this is not a global measure of cognitive dysfunction, but rather targets specific cognitive executive functions frequently affected in MS.

### 2.6. Statistical Methods

All statistical tests were performed using the GraphPad Prism 8 software (GraphPad Software Inc., La Jolla, CA, USA). Shapiro–Wilk test was used to examine the distribution of the data. Mann–Whitney U test was used to compare medians in skewed distributed parameters for unpaired samples and Wilcoxon matched-pairs signed-rank test for paired samples. Correlation analyses were performed with Spearman’s rank correlation and corrected for multiple testing by the Bonferroni method. A *p*-value ≤ 0.05 was considered as statistically significant.

### 2.7. Ethical Statement

The study was reviewed by the appropriate ethics committee of the University of Ulm (approval number 79/2001, approval date 14.11.2001) and was performed in accordance with the ethical standards of the current version of the Declaration of Helsinki. Written informed consent was obtained from all patients participating in this study.

## 3. Results

### 3.1. Serum NfL at Time Points With and Without Active Relapse

**Figure 1:**
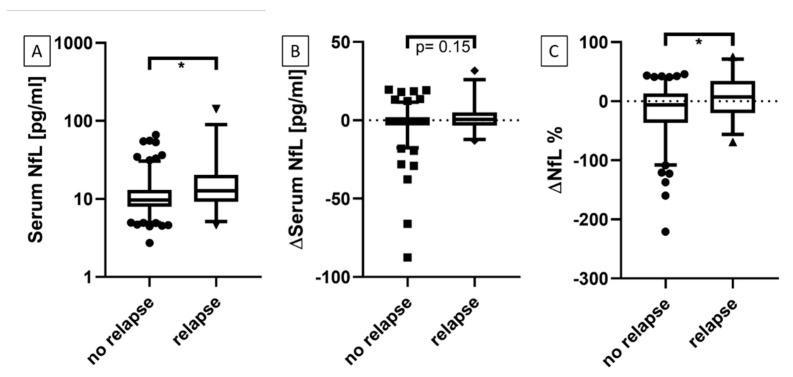
Serum NfL and time points with or without an active relapse. (**A**) Comparison of serum NfL levels during time points with and without an active relapse. (**B**) Comparison of the change of serum NfL between a time point with an active relapse and the previous time point. (**C**) Comparison of the percentage change of serum NfL between a time point with an active relapse and the previous time point. * *p* < 0.05.

We categorized serum NfL levels of all time points accordingly whether an active relapse was present or not. Additionally, the change of serum NfL values at a time point with an active relapse in comparison with the previous time point was determined. Here, the absolute change of serum NfL values (pg/mL) and the percentage change was calculated (tx-tx-1). Significantly higher serum NfL levels were observed for time points with an active relapse compared with time points with no relapse (Figure 1A, *p* < 0.05). This was also true for the percentage change (Figure 1C, *p* < 0.05), but not for the absolute change of serum NfL (Figure 1B, *p* = 0.15).

### 3.2. Serum NfL Levels During Follow-Up Period of 24 Months

#### 3.2.1. Patients with Relapses vs. No Relapse

**Figure 2:**
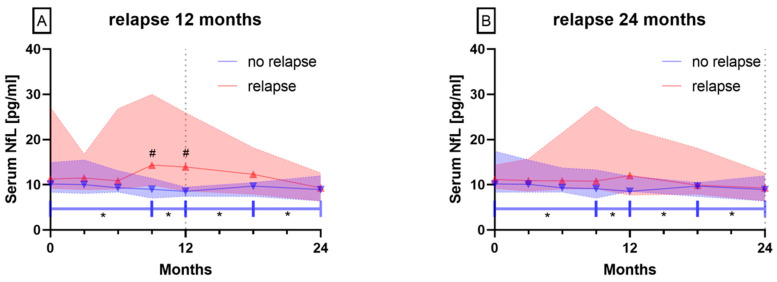
Serum NfL levels over 12 and 24 months in patients with (red triangle facing up) and without relapse (blue triangle facing down) for (**A**) 12 or (**B**) 24 months. Symbols show median values, colored range indicates 95% confidence interval (CI), * *p* < 0.05 for intragroup differences, # *p* < 0.05 for intergroup differences.

In patients with a relapse-free disease course of 12 months and 24 months, serum NfL decreased significantly between baseline and time points 12 months and 18 months and time points 9, 12, 18 and 24 months, respectively (Figure 2A,B, blue triangles facing down, *p* < 0.05). There were no significant differences for baseline and follow-up visits of serum NfL levels in patients with at least one relapse within 12 or 24 months (Figure 2A,B). Furthermore, serum NfL levels in patients with a relapse within 12 months were significantly higher than in patients without a relapse within 12 months at time points 9 and 12 months (Figure 2A, *p* < 0.05).

#### 3.2.2. Patients with EDSS Progression vs. Stable or Improved EDSS

**Figure 3:**
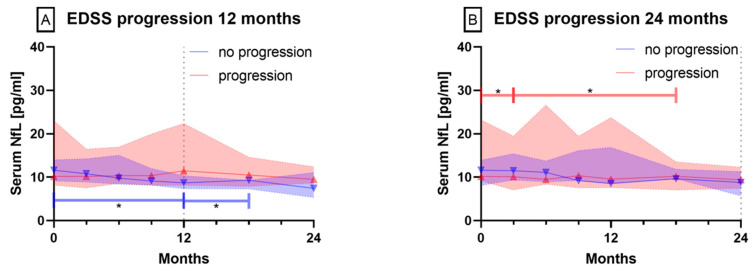
Serum NfL levels over 12 and 24 months in patients with (red triangle up) and without EDSS progression (blue triangle down) within (**A**) 12 or (**B**) 24 months. Symbols show median values, colored range indicates 95% CI, * *p* < 0.05.

In patients showing EDSS progression within 12 months and patients with a stable or improved EDSS for 24 months, no differences concerning their NfL levels were observed. However, in patients with a stable or improving EDSS within 12 months, serum NfL levels decreased significantly between baseline and time points 12 and 18 months (Figure 3A, *p* < 0.05). Considering 24 months of observation, patients with EDSS progression showed serum NfL levels that differed significantly from baseline serum NfL levels after 3 and 18 months (Figure 3B, *p* < 0.05).

### 3.3. Correlation of Serum NfL with

#### 3.3.1. Age

There was no significant correlation of serum NfL and age in our cohort (r < 0.3, *p* > 0.05). However, age effects on serum NfL have been described [9,16]. As we mainly compared longitudinal sNfL values from the same individual over a limited period (24 months), no correction for age was made.

#### 3.3.2. Serum Cytokine Profile in All Patients

We performed correlation analyses for serum NfL and serum IFN-γ, OPN, IL-2, IL-4 and IL-10 and for all time points. There was no significant correlation between serum NfL and the cytokine profile (r < 0.3, *p* > 0.05).

### 3.4. PASAT

**Figure 4:**
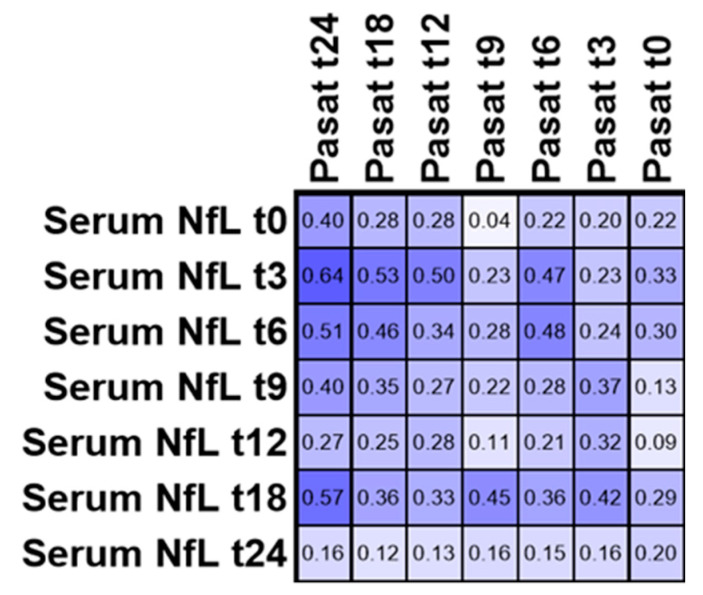
Correlation matrix of Spearman correlation analysis for serum NfL and PASAT. Numbers in the cells are showing the respective Spearman correlation coefficient.

To see whether serum NfL is associated with cognitive decline in MS patients, we performed a correlation analysis of serum NfL and PASAT at all time points (Figure 4). After correcting for multiple testing, a significant correlation between PASAT at month 24 and serum NfL at time points 3 and 18 remained (Spearman *r* = 0.64 and 0.57 and adjusted *p*-value = 0.005 and 0.029, respectively).

### 3.5. EDSS in Patients with Active Disease within 24 Months

**Figure 5:**
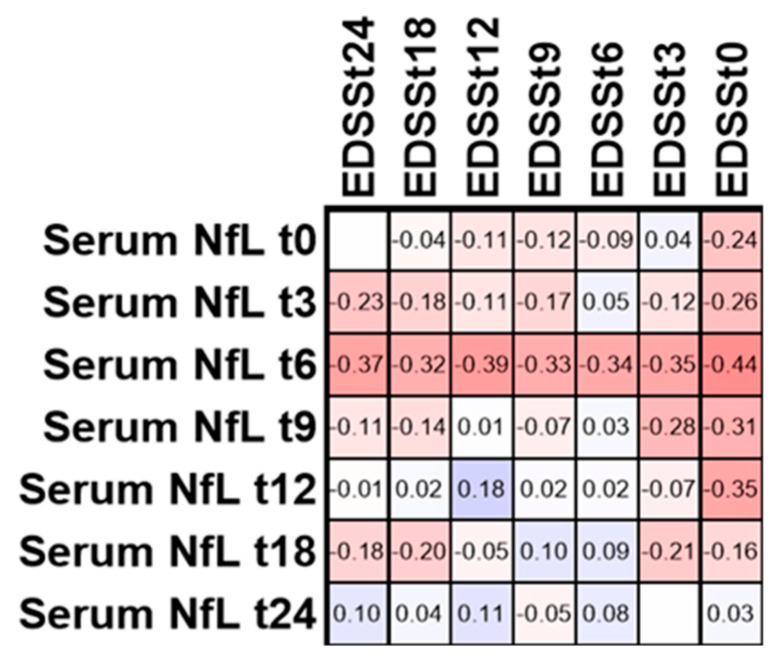
Correlation matrix of Spearman correlation analysis for serum NfL and EDSS. Numbers in the cells are showing the respective Spearman correlation coefficient.

To see whether serum NfL is associated with the disability in MS patients, we performed a correlation analysis of serum NfL and EDSS at all time points (Figure 5).

We did not observe significant correlations for serum NfL and EDSS at any time point in the group with active disease (at least one relapse) within 24 months.

### 3.6. Individual Serum NfL Courses in Patients Treated with Glatiramer Acetate

**Figure 6:**
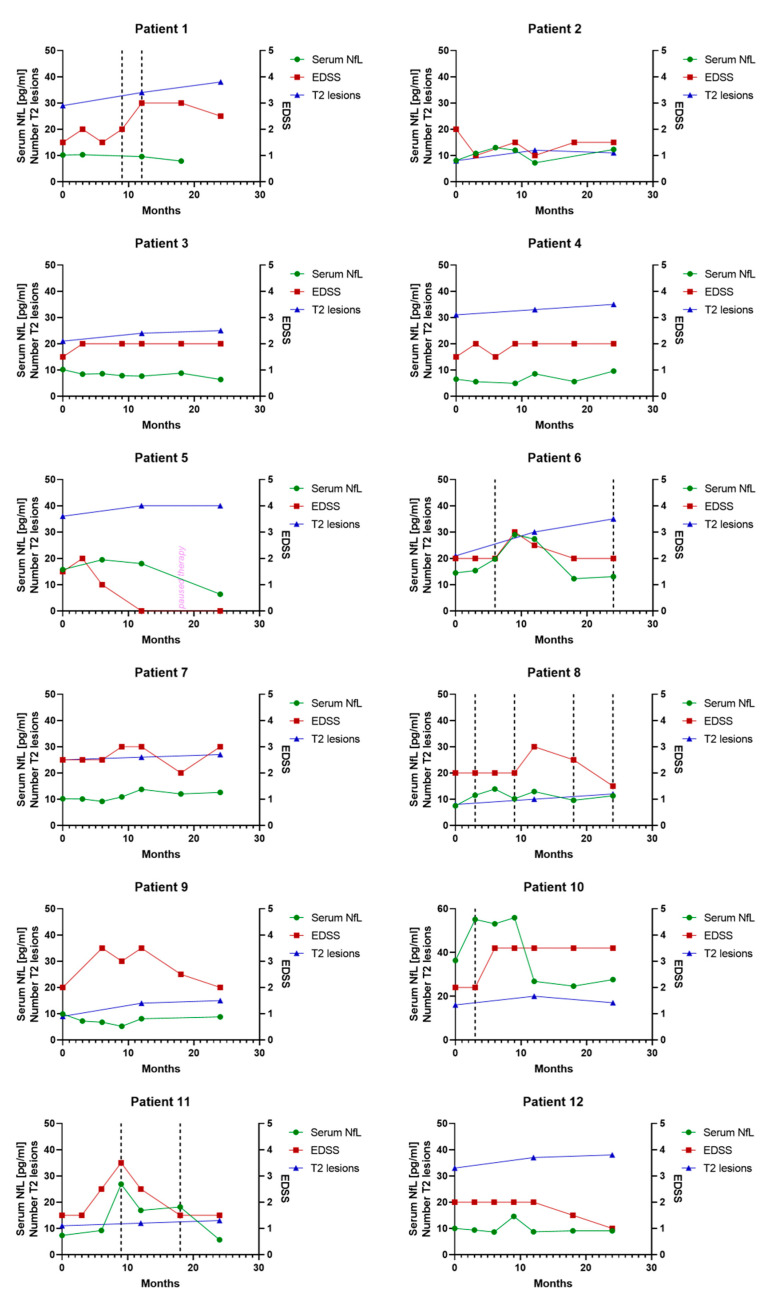

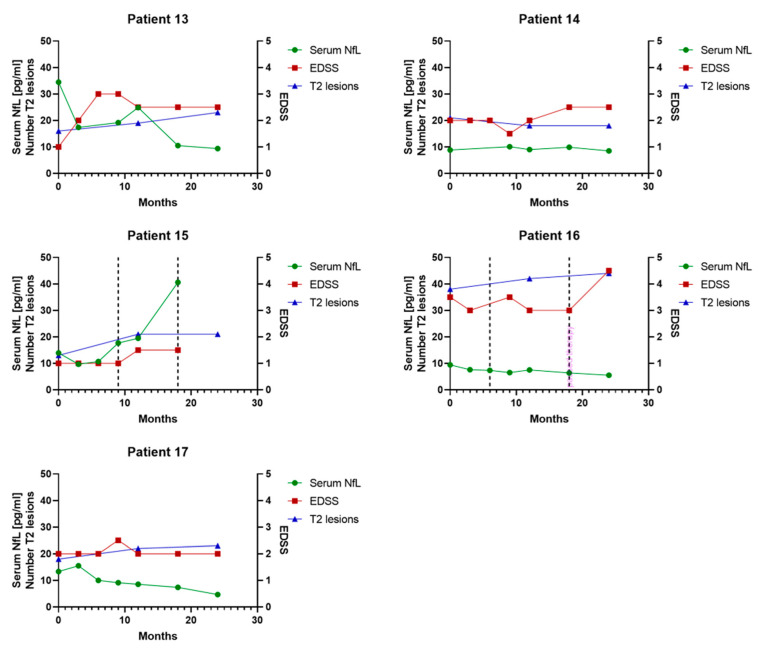
Illustration of individual serum NfL courses over 24 months in patients with initiation of disease-modifying treatment with glatiramer acetate after baseline. Green circles, red squares and blue triangles show serum NfL values (left y-axis), EDSS (right y-axis) and the number of T2 lesions (left y-axis), respectively. Vertical dashed lines show events of clinical activity in the form of a relapse at this time point.

The serum NfL courses of all patients treated with glatiramer acetate with available MRI scans (T2 lesions) are illustrated. Additionally, for every available time point, the EDSS and occurred relapses are shown (Figure 6).

## 4. Discussion

Neurodegeneration and axonal loss are major hallmarks of MS [2]. NfL has been extensively investigated as a biomarker for those molecular processes [6,9,11]. Initially NfL was exclusively analyzed in CSF, but with improved analytical sensitivity, serum analyses became possible as well [17]. Serum NfL shows a good correlation with CSF level and thereby offers a window to monitor axonal loss in MS patients consecutively [18,22]. Therefore, numerous studies including serum NfL in MS are available and it is used frequently in clinical trials [9,11,23,30]. However, longitudinal serum NfL assessments are scarce [26,27], especially in individual MS patients before and after initiation of first-line therapies. For this purpose, we aimed at characterizing the influence of those therapies on serum NfL levels.

Our data suggested that serum NfL may be suitable as a marker for therapy responsiveness based on the following findings: (a) sNfL levels stayed at a consistent low level or even dropped significantly in relapse-free patients over time and (b) sNfL levels after 9 and 12 months were significantly lower in patients without relapse within 12 months compared with patients suffering from a relapse during this time period.

However, we want to point out that most MS patients in the early phase of the disease, which is the case for most of our patients, show serum NfL levels that are within a normal age-adjusted range [16].

Furthermore, our data showed that serum NfL levels were associated with relapses as they were higher in time points with a present relapse compared with non-relapse time points.

The individual serum NfL courses showed that effects that were seen on a group basis did not always hold for every individual. Although serum NfL levels increased during the event of a relapse and decreased after high-dose corticosteroid therapy in most patients, there were exceptions (e.g., patient 16). More consistently, in our cohort, we observed that serum NfL levels stayed at a constant low level in therapy-responsive patients, which might be helpful in therapy monitoring of patients treated with first-line therapies. Our data suggested that this effect can be seen after 9 months. Whereas a sampling interval of 3 or even 6 months seems appropriate in patients without disease activity, other studies of highly active and more severely affected patients suggest a sampling interval and serum NfL testing every month [26]. We did not observe a positive correlation between serum NfL and EDSS for all time points, which is not surprising as this was also not seen in other studies [31] or only described in larger cohorts and with patients more severely affected by the disease and accordingly with higher EDSS [22,32]. The same was true for the correlation of serum NfL and PASAT as cognitive functions are only mildly affected in the early phase of the disease [33]. Even though, in a previous study, we observed an association of a more active disease course with higher levels of pro-inflammatory cytokines and lower levels of anti-inflammatory cytokines in a subpopulation of our study cohort [29], there was no correlation of serum NfL with any of the observed cytokines in the present study.

We also want to discuss the shortcomings of this study. As this was a retrospective analysis of serum NfL in a prospectively collected cohort, pre-analytical effects on serum NfL outcomes must be considered as samples were stored for more than 10 years. However, the observed values were in the same range as those of comparable patients [30,34,35] and of particular interest as no other therapies were available at this time and thereby we were able to monitor long-term outcomes of serum NfL in this specific study population. We can also not completely rule out spontaneous processes or regression that influences serum NfL (sNfL) levels, as we did not include untreated, stable MS patients. As this was an explorative study, these findings need to be confirmed in independent studies and it is desirable to have more detailed MRI data (e.g., number of gadolinium-enhanced lesions, atrophy, etc.) and complete data sets for every patient in those future studies because, for example, T1-hypointense lesions explain the severity of clinical disability better than T2-hyperintense white matter lesions and gadolinium-enhancing lesions correlate better with active disease status. Missing correlation with EDSS was similar to previous findings [31]. However, we were also unable to detect any correlations with the analyzed cytokines. This might be due to the small sample size or that inflammatory processes were either not present in patients or not displayed in the serum of those patients.

Monitoring of subclinical disease activity using MRI is an established procedure in the care of MS patients. Due to the method’s invasiveness, this is not possible for CSF examination, although CSF parameters are appropriate to reflect intrathecal inflammatory processes. Serum NfL appears to be a promising marker for monitoring subclinical disease activity, as demonstrated in this cohort with longitudinal data collection under the same therapy over 24 months. However, this effect may not be seen in every patient as shown in our single-patient illustrations. In a heterogeneous disease like MS, a single biomarker is not sufficient to completely monitor and evaluate therapy efficacy. For this reason, all available information, clinical and paraclinical, should be gathered and taken into account for clinical decision making.

In summary, our study presents the first results on the effect of first-line therapies on serum NfL levels in mildly affected MS patients over 24 months. Here, serum NfL seems especially helpful in detecting therapy-responsive patients, but we also want to address the need for identifying factors that might influence serum NfL values. Among others, this includes processes involved in the transport of NfL from the CSF into serum as well as NfL clearance. The more we know about non-disease-related mechanisms that affect serum NfL, the better we can model serum NfL courses and identify real changes that are caused by pathological processes.

## Figures and Tables

**Table 1:** Patients’ characteristics.

Characteristics	Median Values with IQR, *n* = 34
Age	33 (29–40)
EDSS baseline	1.5 (1.0–2.0)
Serum NfL baseline (pg/mL)	10.2 (8.4–14.7)
Relapse within 12 months (n)	14
Relapse within 24 months (n)	16
Treatment after baseline	
Glatiramer acetate	20
Interferon-beta	12
No disease-modifying therapy	2

EDSS = Expanded Disability Status Scale; NfL = Neurofilament light chain; IQR = Interquartile range.

**Table 2:** Study schedule and number of available data.

	Baseline	3 Months	6 Months	9 Months	12 Months	18 Months	24 Months
Clinical assessment	34	32	32	32	33	34	34
EDSS	31	30	27	30	32	32	32
Serum NfL	34	29	29	32	33	31	24
MRI (T2 lesion load)	17				17		17
Serum cytokine profile	29	29	29	29	29	29	29
PASAT	32	32	31	30	32	30	31

EDSS, Expanded Disability Status Scale; PASAT, Paced Auditory Serial Addition Test; NfL, neurofilament light chain; MRI, magnetic resonance imaging.
